# Late-onset cblC defect: clinical, biochemical and molecular analysis

**DOI:** 10.1186/s13023-023-02890-4

**Published:** 2023-09-28

**Authors:** Si Ding, Shiying Ling, Lili Liang, Wenjuan Qiu, Huiwen Zhang, Ting Chen, Xia Zhan, Feng Xu, Xuefan Gu, Lianshu Han

**Affiliations:** Department of Pediatric Endocrinology and Genetic Metabolism, Xinhua Hospital, Shanghai Institute of Pediatric Research, Shanghai Jiao Tong University School of Medicine, 1665 KongJiang Road, Shanghai, 200092 China

**Keywords:** cblC defect, Late-onset, Methylmalonic acidemia and homocystinuria, Neuropsychiatric symptoms, Prognosis

## Abstract

**Background:**

cblC defect is the most common type of methylmalonic acidemia in China. Patients with late-onset form (>1 year) are often misdiagnosed due to heterogeneous symptoms. This study aimed to describe clinical characteristics and evaluate long-term outcomes of Chinese patients with late-onset cblC defect.

**Methods:**

A total of 85 patients with late-onset cblC defect were enrolled. Clinical data, including manifestations, metabolites, molecular diagnosis, treatment and outcome, were summarized and analyzed.

**Results:**

The age of onset ranged from 2 to 32.8 years old (median age 8.6 years, mean age 9.4 years). The time between first symptoms and diagnosis ranged from a few days to 20 years (median time 2 months, mean time 20.7 months). Neuropsychiatric symptoms were presented as first symptoms in 68.2% of cases, which were observed frequently in schoolchildren or adolescents. Renal involvement and cardiovascular disease were observed in 20% and 8.2% of cases, respectively, which occurred with the highest prevalence in preschool children. Besides the initial symptoms, the disease progressed in most patients and cognitive decline became the most frequent symptom overall. The levels of propionylcarnitine, propionylcarnitine / acetylcarnitine ratio, methylmalonic acid, methylcitric acid and homocysteine, were decreased remarkably after treatment (*P*<0.001). Twenty-four different mutations of *MMACHC* were identified in 78 patients, two of which were novel. The c.482G>A variant was the most frequent mutated allele in this cohort (25%). Except for 16 patients who recovered completely, the remaining patients were still left with varying degrees of sequelae in a long-term follow-up. The available data from 76 cases were analyzed by univariate analysis and multivariate logistic regression analysis, and the results showed that the time from onset to diagnosis (OR = 1.025, *P* = 0. 024) was independent risk factors for poor outcomes.

**Conclusions:**

The diagnosis of late-onset cblC defect is often delayed due to poor awareness of its various and nonspecific symptoms, thus having an adverse effect on the prognosis. It should be considered in patients with unexplained neuropsychiatric and other conditions such as renal involvement, cardiovascular diseases or even multiple organ damage. The c.482G>A variant shows the highest frequency in these patients. Prompt treatment appears to be beneficial.

**Supplementary Information:**

The online version contains supplementary material available at 10.1186/s13023-023-02890-4.

## Introduction

Cobalamin C defect (cblC, OMIM 277,400), accounting for 70% of cases with methylmalonic acidemia (MMA), is the most common disorder of organic acid metabolism in China. This disorder is caused by mutations in the *MMACHC* gene, leading to the impaired conversion of cobalamin into its two metabolically active forms, adenosylcobalamin and methylcobalamin. They are essential cofactors for the conversion of homocysteine (HCY) into methionine in the cytosol and methylmalonyl-CoA into succinyl-CoA in mitochondria, respectively. Their deficiency results in the accumulation of HCY and methylmalonic acid accompanied by normal or decreased methionine levels [1, 2]. According to a recent systematic literature review, the detection rate of MMA (all types) was 1/126,582, 1/89,286, 1/81,967, and 1/16,556 in Asia-Pacific, Europe, North America and the Middle East and North Africa (MENA) regions, respectively [3]. The incidence of MMA in China varies from region to region, which was reported to be 1/5589 in Shandong Jining district [4], 1/6032 in Henan province [5], 1/16,833 in Jiangsu Xuzhou district [6], 1/38,667 in Shanghai [7]and 1/46,531 in Zhejiang province [8].

The cblC defect is inherited in an autosomal recessive pattern. The age of onset ranges from the prenatal to adult stage and clinical presentation can vary considerably, ranging from a mild, potentially asymptomatic phenotype to an acute or chronic form with disease progression, which is at risk of disability or even life-threatening [9–12]. Based on the age of onset, it can be classified into two distinct phenotypes: early onset and late onset [2]. Patients with early onset, presenting symptoms in the first year of life, show severe clinical manifestations, such as feeding difficulties, growth retardation, lethargy, hypotonia, neurological, ophthalmological and hematological complications. Patients with late onset can present symptoms at any time after one year of age and they can be easily misdiagnosed or missed due to the heterogeneous clinical manifestations, encompassing neurological, psychiatric, renal and thromboembolic symptoms [13–15]. Early diagnosis and prompt treatment may prevent symptoms or ameliorate the disease course [16].

In the present study, we performed a detailed retrospective chart review of clinical data in 85 patients with late-onset cblC defect. The aim of this research was to investigate the clinical, biochemical and molecular characteristics of late- onset cblC defect and evaluate their long-term outcomes.

## Methods

### Patients

A total of 85 patients with late-onset cblC defect were recruited at our center between 2010 and 2022. All patients were from unrelated families, except for five siblings P3 and P4, P9 and P23, P42 and P43, P79 and P80 as well as P82 and P83. Written informed consent was obtained from the parents or legal guardians of the study participants. This study was approved by the Ethics Committee of Xinhua Hospital (approval No. XHEC-D-2023-058).

### Metabolite detection

Blood levels of acylcarnitines, including propionylcarnitine (C3) and acetylcarnitine (C2) as well as amino acids were detected by tandem mass spectrometry (MS/MS; Applied Biosystems, API 4000, California, United State) on dried blood filter papers. The ratios of C3/C2 were calculated at the same time. Urinary organic acids including methylmalonic acid and methylcitric acid (MCA) were measured by gas chromatography-mass spectrometry (GC/MS; Shimadzu Limited, QP2010, Kyoto, Japan). Blood HCY were measured by fluorescence polarization immunoassay.

### Mutation analysis

Genomic DNA was extracted from peripheral blood samples. Gene test was performed by Sanger or next generation sequencing. Reference sequences *MMACHC* (NM_015506.2) were obtained from NCBI GENEBANK to identify mutations. The ClinVar database, the HGMD database, and the previous literatures were used to identify whether the mutations had been reported. The pathogenicity of novel variants was evaluated based on the American College of Medical Genetics and Genomics (ACMG) standards and guidelines. The potential pathogenicity of mutations was predicted by Mutation Taster, PolyPhen-2, Provean and SIFT software.

### Treatment

Personalized treatment strategies were given to patients with late-onset cblC defect as soon as it was diagnosed. Intramuscular hydroxyl cobalamin administration was the primary treatment, at a dose of 10 mg/day. In addition, oral administration of L-carnitine (50–100 mg/kg/day), betaine (50–100 mg/kg/day) and folic acid (5–10 mg/day) were also prescribed. The long-term treatment was adjusted depending on the condition of individual patients [16, 17]. Symptomatic treatment was performed for patients with renal disease, cardiovascular disease and other complications.

### Follow-up and outcome evaluation

Patients were followed up every 3–6 months after diagnosis. The content included recent treatment strategies, condition changes, growth and development. Amino acids, acylcarnitines and HCY levels in blood and organic acids levels in urine were analyzed regularly. Blood routine examination, liver function and brain magnetic resonance imaging (MRI) were also monitored. According to the basic motor function and language development evaluation method mentioned in the literature [18], patients were divided into two main groups: normal outcome group and poor outcome group. The normal group outcome showed no significant impairments in daily functioning, whereas the poor outcome group presented with deficits, such chronic renal failure and progressive pulmonary arterial hypertension (PAH) and/or delayed attainment of motor and/or speech milestones, such as requiring assistance for walk and being unable to effectively communicate.

### Statistical analyses

Statistical analyses were performed using SPSS 26.0 (IBM Corp., Armonk, New York). Continuous variables are presented as the mean ± SD or median (range). Data that did not significantly deviate from normal distribution were tested using an unpaired two-tailed t-test, and non-normally distributed data were tested using the Mann-Whitney U test. Categorical variables are presented as frequencies and percentages. Chi-squared test or Fisher’s precision probability test were used to compared categorical variables. Multivariate logistic regression analysis was performed using variables with significant differences in univariate analysis. Statistical significance was established at *P*<0.05.

## Results

### Clinical characteristics

**Fig. 1:**
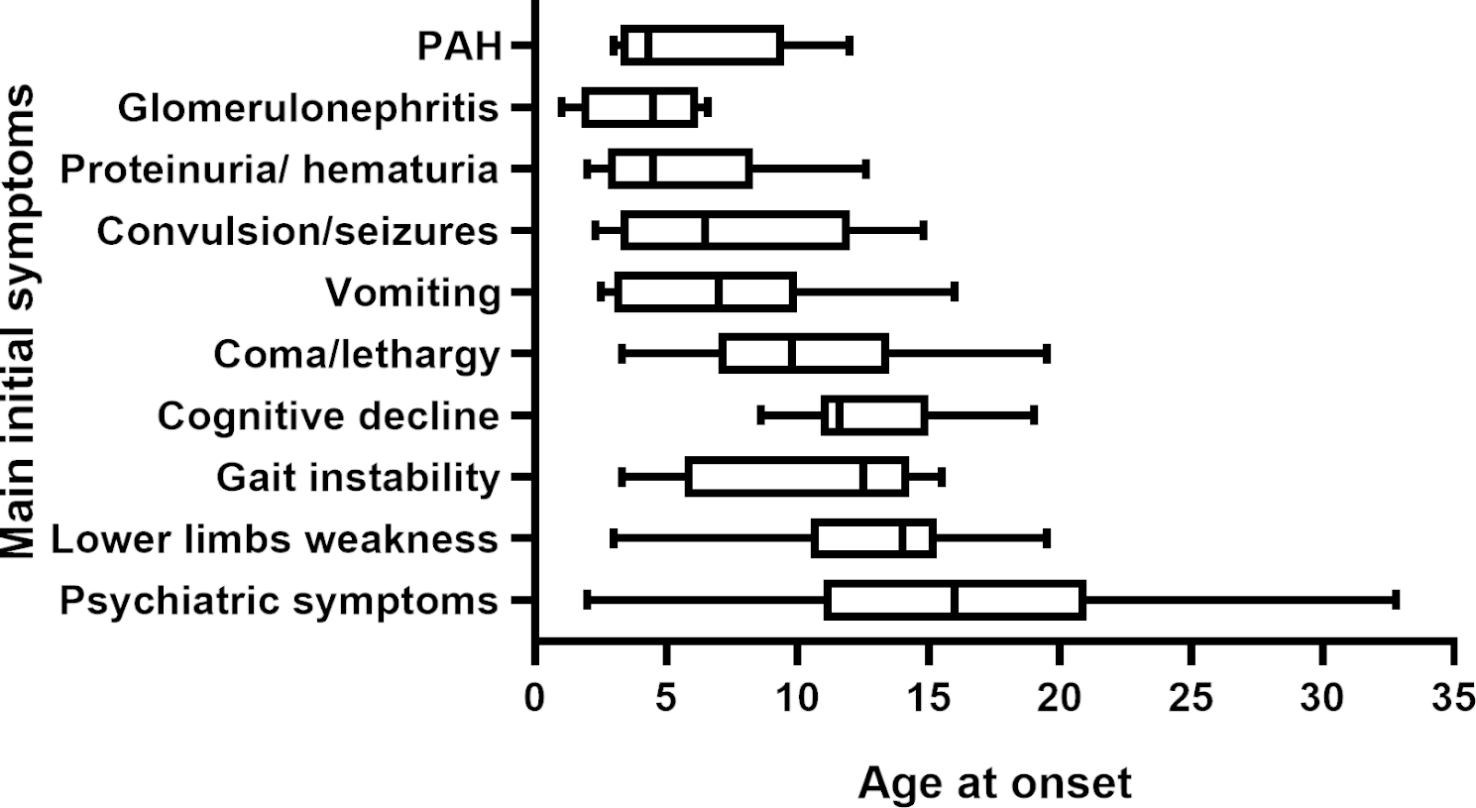
Age at onset for main initial symptoms in 85 patients with the late-onset cblC defect

Detailed patient information is summarized in Supplementary Table S1. In this cohort, 85 patients with late-onset cblC defect were recruited, including 51 males and 34 females. All patients were diagnosed due to disease onset with symptoms. The age of onset ranged from 2 to 32.8 years old (median age 8.6 years, mean age 9.4 years). The time between first symptoms and diagnosis ranged from a few days to 20 years (median time 2 months, mean time 20.7 months). Most patients (58/85, 68.2%) had neuropsychiatric symptoms as first presenting symptoms, followed by renal involvement (17/85, 20.0%), cardiovascular disease (7/85, 8.2%) and metabolic crises (3/85, 3.5%). Detailed age specific patterns of initial symptoms are further depicted in Fig. 1. PAH (median age at onset 4.3, range 3–12 years), proteinuria/hematuria (median age at onset 4.5, range 2.5-8 years) and glomerulopathies (median age at onset 4.5, range 1-6.6 years) were the most frequent symptoms in preschool children. While, in schoolchildren or adolescents, convulsion (median age at onset 6.5, range 2.3–14.8 years), vomiting (median age at onset 7, range 2.5–16 years), lethargy/coma (median age at onset 9.8, range 3.3–19.5 years), cognitive decline (median age at onset 11.6, range 8.6–19 years), gait instability/ataxia (median age at onset 12.5, range 3.3–15.5 years), lower limbs weakness (median age at onset 14, range 3-19.5 years) and psychiatric symptoms (median age at onset 16, range 2.0-32.8 years) were dominant features. And median time from neuropsychiatric symptoms, renal involvement, cardiovascular disease and metabolic crises onset to diagnosis was 2.5 months (mean 15.8, range 0.2–240), 2 months (mean 37.8, range 0.2–149), 4 months (mean 5, range 0.2-146.8) and 1 months (mean 1.7, range 0.5–3.7), respectively.

**Fig. 2:**
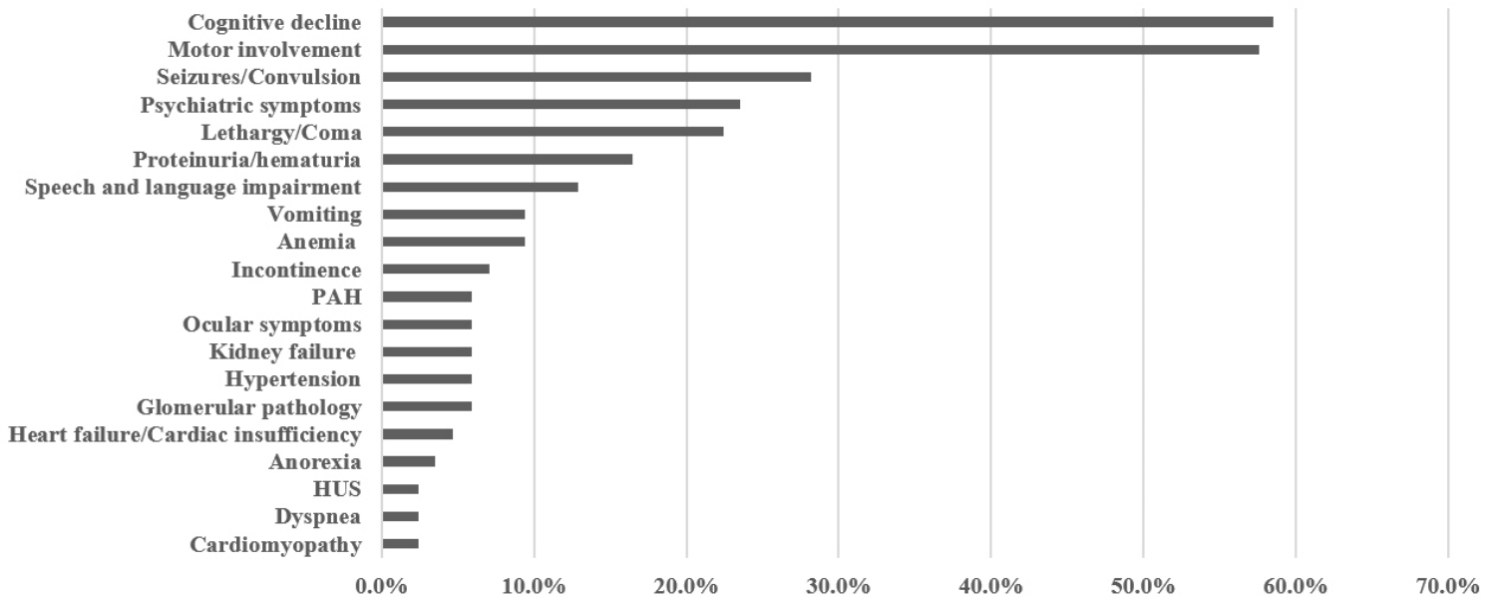
Frequency of overall clinical symptoms in 85 patients with late-onset cblC-type MMA

Besides the first symptoms at onset, disease progressed in overwhelming majority of patients with cblC defect. Figure 2 shows the frequency of overall clinical symptoms in 85 patients with late-onset cblC defect. Overall, neuropsychiatric symptoms were the most common clinical symptoms among our patients, which were present in 80.0% of cases (68 patients). Among them, cognitive decline, manifesting as a decline in school and work performance or sluggish response, was the most frequent clinical manifestation and presented in 58.8% of cases (50 patients) during disease progression. Other neuropsychiatric symptoms, such as motor involvement (encompassing gait instability, ataxia, lower limbs weakness and spastic paraplegia), seizures/convulsion, psychiatric symptoms (encompassing social withdrawal, insomnia, euphoria anxiety, depression and auditory hallucination), lethargy/coma, speech and language impairment, incontinence and visual involvement were present in 57.6% (49 patients), 28.2% (24 patients), 23.5% (20 patients), 22.4% (19 patients),12.9% (11 patients), 7.1% (6 patients) and 5.9% (5 patients) of cases, respectively. Renal involvement was observed in 23.5% (20 patients) of cases, ranging from proteinuria or hematuria (16.5%), glomerular diseases (5.9%), kidney failure (5.9%) and hemolytic uremic syndrome (HUS, 2.4%). Cardiovascular disease, such as PAH (5.9%), heart failure (4.7%) and cardiomyopathy (2.4%), were identified in eight patients. Further, eight patients (9.4%) presented with anemia, three patients (3.5%) had anorexia and three patients (3.5%) showed signs of metabolic crises, such as vomiting, dyspnea and anorexia.

Brain MRI findings were available for 27 patients. Among them, normal brain imaging was present in four patients (14.8%). The most common abnormalities of brain MRI findings were cortical atrophy (10/27, 37.0%), ventricular dilation or sulcal widening (7/27, 25.9%) and periventricular white matter change (4/27, 14.8%). The other findings of brain MRI included hydrocephalus, subacute cerebral infarction, encephalomalacia foci, cerebral venous sinus thrombosis and thinning of the corpus callosum, which was present in single patient.

### Long-term treatment

During the follow-up, all patients followed a normal diet. According to the literature and our clinical experience, a satisfactory metabolic control is obtained in the case that HCY is not exceeding 50 µmol/L [19]. Thus, hydroxyl cobalamin dosages were adjusted depending on the condition of individual patients, with the single doseof 5 to 20 mg each time, once a day to once every three weeks. Up to February 2023, two patients (P47 and P72) were lost of follow-up. 13 patients were only treated with intramuscular hydroxyl cobalamin, discontinuing oral administration. Two patients (P1 and P29) had complete withdrawal voluntarily because of the improvement of symptoms, which occurred 5 and 2 years after treatment, respectively. The remaining 68 patients accepted treatments with intramuscular hydroxyl cobalamin combined with oral drugs, including betaine, L-carnitine and/or folic acid. Further, symptomatic treatment was performed for patients with some complications such as renal and cardiovascular diseases.

### Biochemical characteristics

As the biochemical makers of patients with cblC defect, blood values C3, C3/C2 ratio, HCY and urinary methylmalonic acid and MCA were measured before and after treatment. Since there were some confirmed patients who had begun vitamin B_12_ treatment in other hospitals and then transferred to our clinic, their biochemical results were partly missing. Therefore, we just analyzed specific biochemical results before and after treatment. As is shown in Table 1, the levels of HCY and methylmalonic acid were elevated before treatment, accompanied by increased or normal C3, C3/C2 ratio and MCA. In addition, all the biochemical markers above revealed statistically significant before and after treatment (P<0.001). These data indicated that patients were vitamin B_12_ responsive.

**Table 1:** Comparison of biochemical data of patients with late-onset cblCdefect in blood and urine before and after treatment

	C3(µmol/L)		C3/C2		Methymalonic acid (mmol/mol cr)		MCA (mmol/mol cr)		HCY(µmol/L)	
	n	Median (range)	n	Median (range)	n	Median (range)	n	Median (range)	n	Median (range)
BEFORE TREATMENT	63	6.61 (1.88–35.44)	64	0.52 (0.11–2.68)	60	94.45 (5.50-845.10)	54	1.63 (0.00-16.61)	72	100.00 (36.10-635.60)
AFTER TREATMENT	63	3.20 (0.96–12.29)	64	0.16 (0.03–0.61)	60	6.32 (0.00-78.66)	54	0.39 (0.00-3.63)	72	39.00 (1.37–84.60)
*P* VALUE	＜0.001		＜0.001		＜0.001		＜0.001		＜0.001	
REFERENCE RANGE	0.40-4.00		0.03–0.20		0.00–4.00		0.00-0.70		<15.00	

### Mutation spectrum

**Fig. 3:**
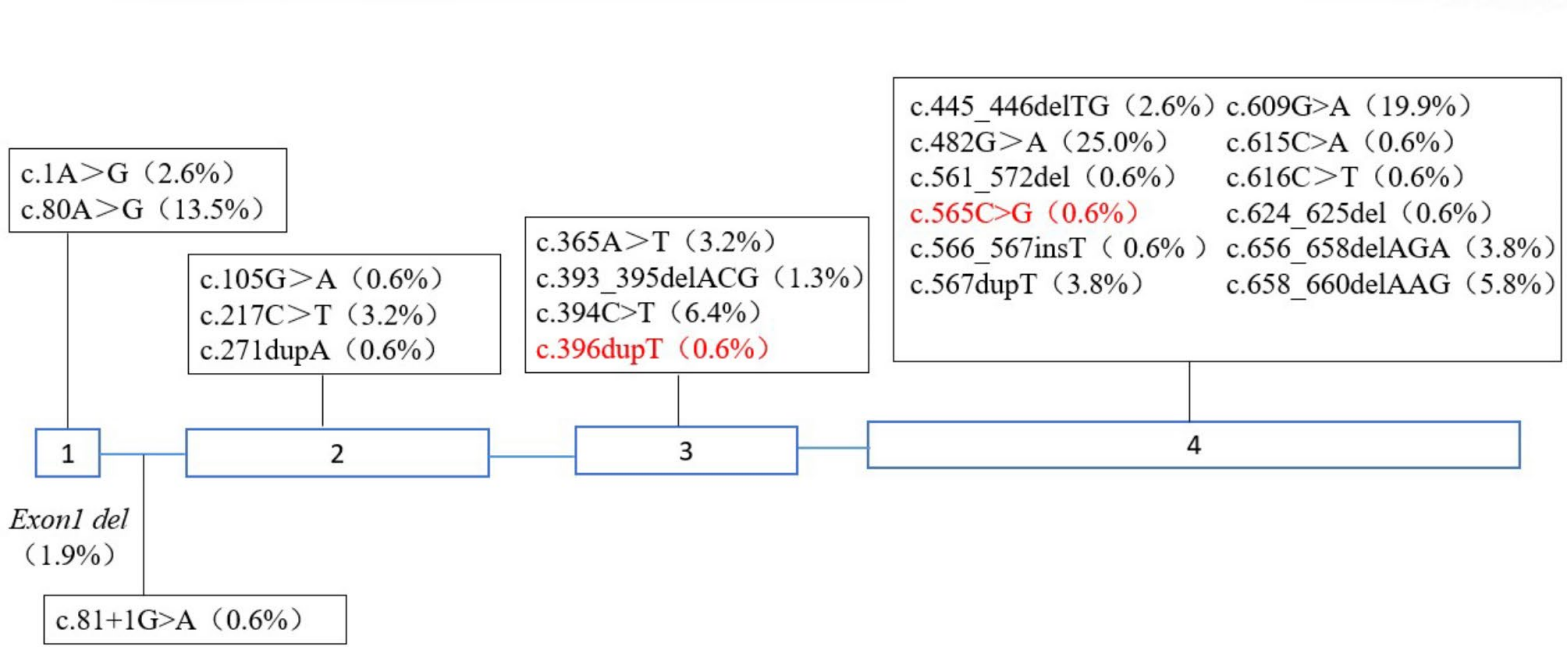
Distribution of the mutations in the *MMACHC *gene in 78 patients with late-onset cblC defect Novel mutations are shown in red.

Molecular analysis was performed on 78 patients. The mutation spectrum of *MMACHC* gene observed in this study is shown in Supplementary Table S1. Five cases (P9, P11, P23, P66 and P75) harbored homozygous variants (two with c.80 A>G, two with c.394 C>T and one with c.482G > A mutation homozygote) and the remaining 73 cases harbored compound heterozygous variants. A total of 24 different variants are associated with late-onset cblC defect in our cohort (Fig. 3). They were distributed throughout *MMACHC* gene from exon 1 to exon 4. The c.482G>A occurred with the highest frequency, followed by c.609G > A, c.80 A > G and c.394 C > T, accounting for 25%, 19.9%,13.5% and 6.4% of all alleles, respectively. Among them, a total of two novel mutations have never been reported before, including c.396dupT and c.565 C>G, which were predicted as disease-causing by Mutation Taster, PolyPhen-2, Provean and SIFT software.

### Clinical outcomes

Up to February 2023, the patients were 4.6–37.2 years old, with a median age of 15 and the median time of follow-up was 4.9 years (mean time 5.1 years, range 0.5-13.4years). Two patients (2.4%) were lost to follow-up (P47 and P72) and the remaining patients were alive.16 patients (18.8%) were healthy. Clinical symptoms improved but not completely recovered in 14 patients (16.5%), with five patients suffering from cardiovascular disease, eight patients manifesting renal involvement and one patient subject to anemia. The remaining 53 patients (62.4%) suffered from poor outcomes, such as movement disorders, recurrent convulsion, intellectual impairment, chronic renal failure and progressive PAH. (Supplementary Table S1)

Univariate analysis and multivariate logistic regression analysis were performed among 76 patients to determine the factors affecting prognosis after excluding patients who were lost follow-up and with missing data. Factors including age at onset, time from onset to diagnosis, initial symptoms and genotype were analyzed. Considering that c.482G > A, c.80 A>G and c.394 C>T were observed frequently in late-onset cases according to our findings and literature [20, 21], the genotype was divided into four groups, c.482G > A, c.80 A>G, c.394 C>T and others. As shown in Table 2, there were significant differences in age at onset, time from onset to diagnosis, neuropsychiatric symptoms or renal involvement as initial symptoms and carrying c.482G>A or c.80 A>G variant between normal outcome groups and poor outcome groups. Multivariate logistic regression analysis was performed using above variables to determine the independent risk factors. The results are listed in Table 3. It showed that the time from onset to diagnosis (OR = 1.025, *P* = 0. 024) was independent risk factors for poor outcomes.

**Table 2:** Comparison of baseline characteristics between patients with normal and poor outcomes

VARIABLE VALUE	NORMAL OUTCOME (n = 30)	POOR OUTCOME (n = 46)	*P* VALUE
**Age at onset (years)**	6.8(2–15)	11(2-32.8)	**0.011**
**Time from onset to diagnosis (months)**	1(0.2–132)	5(0.2–240)	**0.019**
**Initial symptoms**			
Neuropsychiatric symptoms	14(30.3)	37(69.7)	**0.001**
Renal involvement	11(68.8)	5(31.2)	**0.016**
Cardiovascular disease	4(66.7)	2(33.3)	0.205
Metabolic crises	1(33.3)	2(66.7)	1.000
**Nucleotide variant**			
c.482G > A	10(27%)	27(73%)	**0.031**
c.80 A > G	12(66.7%)	6(33.3%)	**0.034**
c.394 C > T	2(25.0%)	6(75.0%)	0.275
Others	6(46.2%)	7(53.8%)	0.955

**Table 3:** Results of logistic regression analysis of factors influencing prognosis of patients with late-onset cblC defect

FACTORS	*OR*	95%CI	*P* VALUE	
**Age at onset (years)**	1.112	0.969–1.276	0.131	
**Time from onset to diagnosis (months)**	1.025	1.003–1.046	**0.024**	
**Initial symptoms**				
Neuropsychiatric symptoms	7.771	0.872–69.211	0.066	
Renal involvement	0.546	0.085-3.500	0.523	
**Nucleotide variant**				
c.482G > A	1.008	0.251–4.053	0.991	
c.80 A > G	2.928	0.338–25.351	0.329	

## Discussion

Approximately 90% of reported patients with cblC defect are severe infantile early onset [9]. While clinical manifestations of late-onset form are quite different from early-onset cases and often complicated with multiple organ damage, thus these patients might be easily misdiagnosed or missed [13, 15, 22]. In the present study, we collected clinical data from 85 patients with late-onset cblC defect, described their clinical, biochemical and molecular characteristics and analyzed prognosis and influencing factors.

In our study, 85 patients were healthy before onset, exhibiting normal development. The initial symptoms varied widely and patterns of these clinical manifestations seem to vary with age. Among them, neuropsychiatric symptoms were the most common manifestations in schoolchildren or adolescents. Renal involvement and cardiovascular disease showed a high prevalence in preschool children and were not observed in adults. Metabolic crises were relatively rare. In agreement with this finding, Huemer et al [15] also found that, HUS and PAH were main presenting symptoms in preschool children. While psychiatric symptoms, cognitive decline, ataxia and myelopathy were frequent in older children or adolescents. And in adults, thrombosis, neuropathy, myelopathy and glomerulopathies were mainly observed. Besides initial symptoms, disease progressed in most patients. Overall, cognitive decline and motor involvement were dominant symptoms, while ocular involvement seems to be rare in late-onset cblC defect. All these findings were correlated with previous studies [13, 22]. This suggests that patients with late-onset cblC defect can present with a wide spectrum of nonspecific symptoms, of which neuropsychiatric symptoms are the most common manifestations and often occur as the initial symptoms. The time between first symptoms and diagnosis ranged widely in our study, the longest of which was up to 20 years. Therefore, late-onset cblC defect is a disease involving multiple systems and organs, and it should be considered when patients have unexplained neuropsychological symptoms, metabolic crises or other organ involvement. And detailed statistical analysis of larger groups of patients may assist in further exploring the relationship with genotypes for other symptoms and the correlation between time from onset to diagnosis and initial symptoms. Despite heterogeneous presenting symptoms, heterogeneous symptoms, metabolite measurement using MS/MS and GC/MS is considered as a necessary method for the diagnosis of MMA. In this study, plasma HCY and urinary methylmalonic acid concentrations in all patients were markedly increasing, which are regarded as useful indicators for the prompt diagnosis of cblC defect [16].

Abnormal brain MRI results also contribute to evaluating the extent of brain injury and prognosis. In our study, cerebral atrophy, periventricular white matter abnormality, ventricular dilation and sulcal widening were the main findings in patients with cblC defect, which was correlated with previously published literature [23, 24]. However, MRI features of MMA are nonspecific and do not permit differentiation from other metabolic diseases and classification of MMA.

The pathogenic mechanisms have not been entirely elucidated at present. One of the probable reasons might be associated with mitochondrial energy metabolism disorders, which are caused by the accumulation of toxic metabolites [2]. The variety of clinical manifestations in patients with combined MMA seem to be more substantial, which may result from high levels of HCY and low concentrations of methionine. It seems to be believed that the synergistic effect of different mechanisms might be responsible for multiple systems and organs involvement, such as direct neurotoxicity causing cell death, oxidant stress and inflammation, the initiation of a cellular cascade of apoptosis, interference with DNA repair system and N-methyl-d-aspartate-mediated mechanisms [25, 26].

Until now, more than 100 different mutations of *MMACHC* have been reported, and c.482G > A, c.80 A>G and c.394 C>T were observed frequently in late-onset cases [20, 21]. From a genetic point of view, late-onset cases tend to be characterized by compound heterozygosity with a milder variant (missense, inframe deletion/ duplication), which seems to retain a residual function. In our study, 93.6% (73/78) of cases harbored compound heterozygous variants. A total of 24 different mutations of *MMACHC* were identified, of which c.396dupT and c.565 C>T were novel variants. Consistent with previous studies, c.482G > A, c.80 A>G and c.394 C>T were observed frequently in our cohort of late-onset cases, ranking first, third and fourth most common alleles, respectively. The c.609G > A variant also occurred frequently in our study. This nonsense mutation results in a pre-mature termination codon, which is predicted to cause a truncated or absent MMACHC protein, thus it is highly prone to the early-onset form [27, 28]. It is notable that all patients with c.609G > A were compound heterozygotes for a missense variant or c.394 C>T variant, except one with a deletion mutation in the * MMACHC* gene. It has been reported that the early-onset allele tends to be underexpressed when compared to the late-onset allele [20]. Therefore, one of the possible reasons might be associated with the lower expression of *MMACHC* transcription in the c.609G > A variant. In addition, c.609G > A was reported to be the most common variant among Chinese population [12]. As a result, its high frequency in late-onset cases might be explained.

At present, the mainstay of therapy for cblC disease is intramuscular hydroxyl cobalamin, supplemented with oral drugs including betaine, L-carnitine and/or folic acid [17, 29]. Most patients in our study were treated with the above therapy. Although there are theoretical reasons for using L-carnitine and folinic acid, no beneficial effect of adjunctive therapy with folic folinic acid and L-carnitine has been reported in some cases [30, 31]. In 13 patients who only received treatment with hydroxyl cobalamin in the present study, 10 had a c.482G > A variant, and all of their clinical symptoms as well as biochemical data were stable during follow-up. Further data need to determine the effect of only hydroxyl cobalamin administration.

Although late-onset cases are less life-threatening compared to early-onset cases, lack of diagnosis or even late initiation of treatment can lead to a less favorable course with significant morbidity or even death [32]. In this study, all patients who had follow-up survived, of whom 30 patients with normal outcomes, showed no significant impairments in daily functioning, while 53 patients suffered from poor outcomes during the follow-up. This suggests that late-onset cblC defect seems to have a high disability rate. The results of multivariate logistic regression analysis indicated that time from onset to diagnosis is the independent risk factor affecting prognosis. Thus, early diagnosis and timely treatment is essential for prognosis improvement. Luckily, MMA has been included in expanded newborn screening in several countries and cost-effectiveness of using MS/MS in NBS have been reported [33, 34]. However, false positive and negative results should be considered [35–37]. Mutation analysis is essential for a definite diagnosis. Thus, combined biochemical and mutation analysis is recommended to achieve an early and precise diagnosis.

## Conclusion

In conclusion, late-onset cblC defect is difficult to identify due to a wide diversity of symptoms, of which various neuropsychiatric symptoms are the most common symptoms. This disease should be considered in unexplained cases, especially in patients with neuropsychiatric, renal and cardiovascular diseases or even multiple organ damage. The c.482G > A variant is the most frequent variant in late-onset cases. Raising awareness for this disorder helps to improve outcome, accompanied by prompt treatment.

### Electronic supplementary material

Below is the link to the electronic supplementary material.

Supplementary Material 1

## Data Availability

All data generated or analyzed during this study are included in this published article and its supplementary information files.

## Abbreviations

cblC: cobalamin C
MMA: Methylmalonic acidemia
HCY: Homocysteine
MENA: Middle East and North Africa
C3: propionylcarnitine
C2: Acetylcarnitine
C3/C2: propionylcarnitine to acetylcarnitine ratio. GC/MS:Chromatography/mass spectrometry
MS/MS: Tandem mass spectrometry
MCA: Methylcitric acid
ACMG: American College of Medical Genetics and Genomics
MRI: Magnetic resonance imaging
HUS: Hemolytic uremic syndrome
PAH: Pulmonary arterial hypertension
